# Atrio-Ventricular Block by Legionella Disease

**DOI:** 10.7759/cureus.33498

**Published:** 2023-01-08

**Authors:** Kyaw Oo, May Thiri Lwin, Jo Porter

**Affiliations:** 1 Internal Medicine, North West Anglia NHS Foundation Trust, Peterborough, GBR; 2 Cardiology, North West Anglia NHS Foundation Trust, Peterborough, GBR

**Keywords:** legionella infection, legionella pneumonia, community-acquired pneumonia (cap), second-degree heart block, atypical pneumonia, extrapulmonary legionella, mobitz type 2 av block, atrioventricular conduction block

## Abstract

Although Legionnaires’ disease mainly affects the lungs, it can also present with other systemic involvement, including rare cardiac manifestations. Recognised presentations are endocarditis, myocarditis, pericarditis, and pericardial effusion. A 72-year-old British man presented with a six-day history of dry cough and a four-day history of fever during the peak of the COVID-19 pandemic. His electrocardiogram showed Mobitz type II atrio-ventricular block. Although all the cultures were negative, the chest X-ray demonstrated COVID-19 infection-like features. With high clinical suspicions and chest X-ray features, the polymerase chain reaction of the COVID tests was repeated three times and all were negative. He had a positive urinary Legionella antigen, and his bradycardia and heart block improved after treatment with amoxicillin/clavulanic acid, and clarithromycin. As the electrocardiogram showed Mobitz type II, a permanent pacemaker was implanted. The follow-up pacemaker check showed that he still required active pacing.

## Introduction

In 2020, 295 cases of Legionnaires’ disease were reported to the National Surveillance Scheme with an average of 400 cases yearly from 2017 to 2019 [1]. Although Legionella disease is a systemic infection, it commonly manifests as pneumonia. However, when extrapulmonary manifestation does occur, its commonest site of infection is the heart. Even so, it rarely presents conduction defects. We find this case more unique due to the involvement of atrio-ventricular node and its conduction rather than the sino-atrial node.

## Case presentation

Medical history and demographics

A 72-year-old independent Caucasian man presented to the emergency department with a six-day history of dry cough and a four-day history of fever during the peak of the COVID-19 pandemic. His only past medical history was hypertension, for which he was on Lisinopril 5 mg once a day. His previous ECG from a year ago was a normal sinus rhythm. He denied prodromal symptoms such as vomiting and diarrhoea. There were no cardiac symptoms such as presyncope, syncope, chest pain, or palpitation. Moreover, he denied recent travel or COVID-19 contacts.

On presentation to the acute medical unit, he was afebrile with a blood pressure of 145/87 and a heart rate of 84/min but was tachypnoeic and in respiratory distress with a respiratory rate of 21/min and hypoxia (oxygen saturation of 94% on 3 L of oxygen). Auscultation of the lungs revealed equal air entry bilaterally with very few fine bibasal crackles. The jugular venous pressure was not elevated and there was no calf tenderness or swelling. Given the COVID-19 pandemic, this became the presumptive diagnosis.

Investigations

Initial laboratory results revealed that he had neutrophil leucocytosis and lymphopenia with raised D-dimer and C-reactive protein (Table 1). Arterial blood gas on room air on admission showed type 1 respiratory failure (Table 2).

**Table 1:** Initial blood tests and results

Blood test	Value	Reference range
Haemoglobin (g/L)	137	13.5–17.5
White cell count (10^9^/L)	10.6	04–11
Neutrophil (10^9^/L)	9.1	2.0–7.5
Lymphocyte (10^9^/L)	0.8	1.5–4.5
Platelet (10^9^/L)	451	150–400
D-dimer (ng/ml)	1135	<243
Sodium (mmol/L)	132	135–155
Thyroid-stimulating hormone (mU/L)	0.96	0.4–4
C-reactive protein (mg/L)	347	<5
Pro brain natriuretic peptide (pg/ml)	354	<300
Troponin (ng/L)	26	<12

**Table 2:** Arterial blood gas on room air

Anaylse	Value	Refernce Range
pH	7.41	7.35–7.45
pCO_2_ (kPa)	4.75	4.5–6.0
pO_2_ (kPa)	9.22	>11

**Figure 1:**
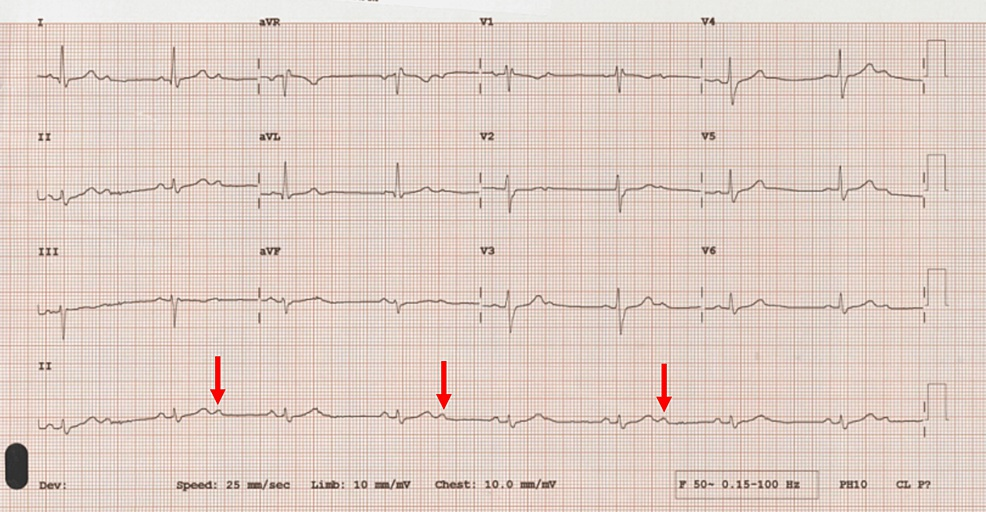
Electrocardiogram Atrio-ventricular block (2:1); non-conducted 'p' wave (red arrow)

**Figure 2:**
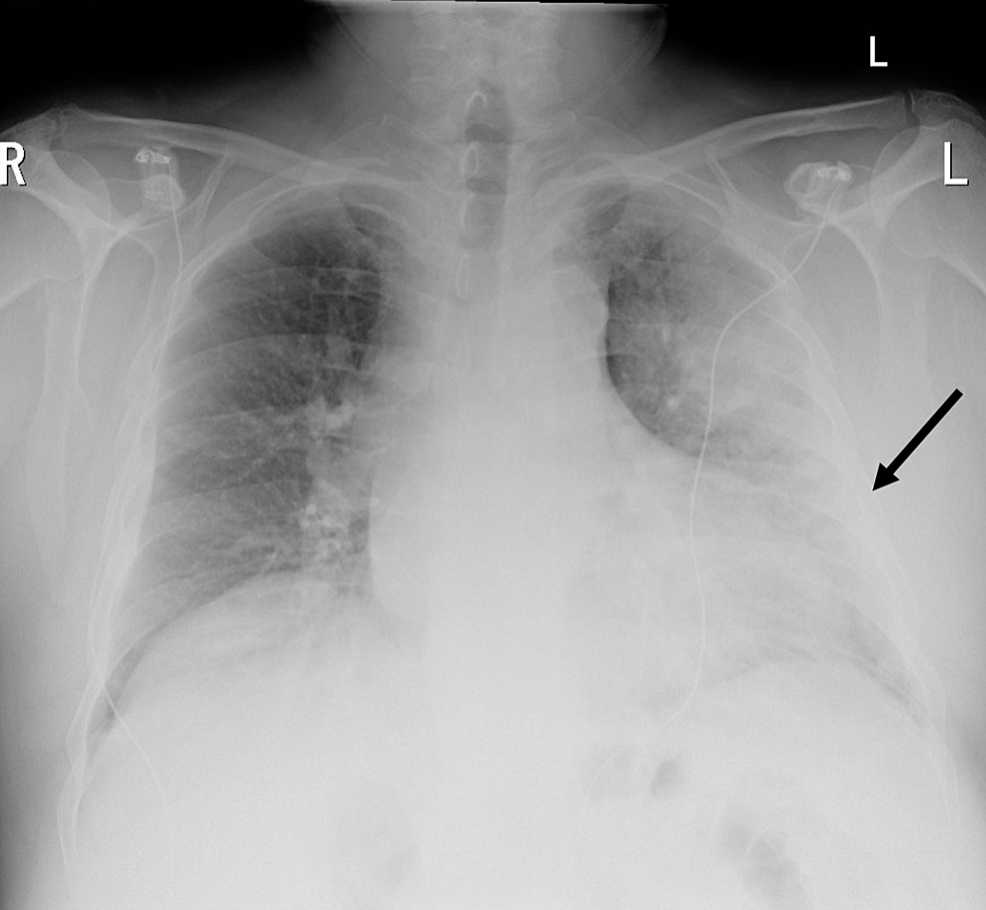
Chest X-ray Consolidation of left lung (black arrow)

The electrocardiogram on admission showed 2:1 atrioventricular block with right bundle branch block (Figure 1) and, unfortunately, no baseline to compare with. Chest X-ray showed extensive airspace shadowing on the left but the right lung was essentially clear (Figure 2).

**Figure 3:**
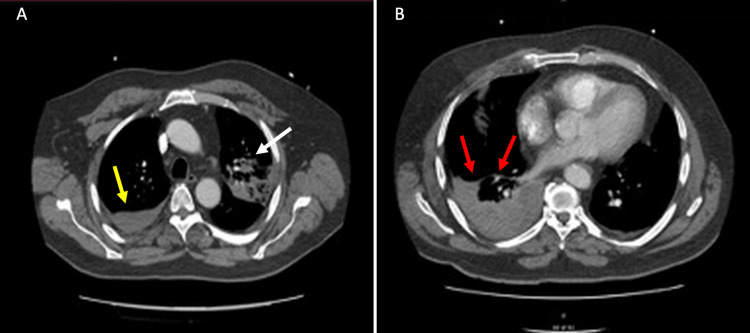
Computed tomography scan of the chest (A) Consolidation of left upper lobe (white arrow) with pleural effusion on the right side of the lung (yellow arrow), and (B) mild pleural effusion on the right lung field (red arrow)

Although the suspicion of pulmonary embolism was raised due to high D-dimer, computed tomography of the pulmonary angiogram excluded it but confirmed upper lobe consolidation on the left lung, suggesting infection. There was a mild right pleural effusion (Figure 3).

Community-acquired pneumonia screening and chlamydia testing were negative, with blood and sputum cultures showing no growth. Urinary Legionella antigen returned positive later. Repeated viral studies for COVID-19 infection were negative. In addition, the polymerase chain reaction results for influenza A, B, avian flu, and the immunoglobulin G antibody to pigeons were negative.

The echocardiogram showed good left and right ventricular systolic function with an ejection fraction of above 60% and did not show regional wall motion abnormalities. As there will be no benefit from the management plan, cardiac magnetic resonance imaging was not performed.

Treatment

The initial treatment for the possible COVID-19 infection included oral steroids and injection of co-amoxiclav and oral clarithromycin for a possible secondary bacterial chest infection. After receiving the computed tomography pulmonary angiogram report and a negative COVID-19 result, steroids and antibiotics were stopped, and doxycycline was started to treat atypical pneumonia. On day 2 of doxycycline, his urinary Legionella antigen was positive. Therefore, he was treated with oral doxycycline for 14 days according to guidelines. Permanent pacemaker implantation was planned once the infection had settled.

Outcome and follow-up

A permanent pacemaker was later implanted as an outpatient for Mobitz type II heart block. At the follow-up, he was actively pacing. A follow-up chest X-ray showed that there was an improvement in consolidation changes, although the small right-sided pleural effusion remained unchanged. He has been followed up by the respiratory team for further investigations of his pleural effusion.

## Discussion

While several infectious diseases are associated with cardiac conduction abnormalities, there are only two case reports describing Legionella-induced complete heart block and sinoatrial block, respectively [2]. Conduction disturbance appears to be more common in paediatric populations.

Although the aerosolization of contaminated water is the known route of transmission for Legionella, there was no obvious exposure and no relevant travel history. The source of infection in this case is unclear. According to Public Health England, the culture of sputum is the gold standard for the investigation of legionella, but urinary antigen testing produces faster results, leading to lower mortality [3]. Doing a urinary antigen test alone is acceptable by Public Health England to achieve the diagnosis with 70% sensitivity and nearly 100% specificity [4].

The prognosis of Legionella-associated cardiac conduction problems remains unknown. According to our case and other related case reports, it is temporary with rapid diagnostics and appropriate therapy. At the subsequent follow-up, our patient had paced 15% in the atrium and 15% in the ventricle. Even though pacemaker insertion was not necessary in other case reports where patients received aggressive supportive care with transcutaneous and transvenous pacing, our patient did require permanent pacemaker implantation, with presumably some underlying conduction abnormality exacerbated by the Legionella infection [5,6].

## Conclusions

In this article, we present the case of a 72-year-old male with symptoms of respiratory infection and Mobitz type II atrio-ventricular block, initially treated for suspected SARS-CoV-2 at the peak of the COVID-19 infection. On further investigations, it was concluded that cardiac complication was due to co-current Legionella infection.

We strongly suggest that in cases of new-onset cardiac conduction disturbance in the setting of community-acquired pneumonia, clinicians should consider Legionella infection as a differential diagnosis. Prompt recognition and treatment of Legionella are crucial to minimise secondary complications such as cardiac involvement and prevent the reversible infectious heart block.
